# Chiral Phosphinate Degradation by the *Fusarium* Species: Scope and Limitation of the Process

**DOI:** 10.1155/2013/927361

**Published:** 2013-11-10

**Authors:** Natalia Kmiecik, Magdalena Klimek-Ochab, Małgorzata Brzezińska-Rodak, Paulina Majewska, Ewa Żymańczyk-Duda

**Affiliations:** Department of Bioorganic Chemistry, Wroclaw University of Technology, Wybrzeże Wyspiańskiego 27, 50-370 Wroclaw, Poland

## Abstract

Biodegradable capacities of fungal strains of *Fusarium oxysporum* (DSMZ 2018) and *Fusarium culmorum* (DSMZ 1094) were tested towards racemic mixture of chiral 2-hydroxy-2-(ethoxyphenylphosphinyl) acetic acid—a compound with two stereogenic centres. The effectiveness of decomposition was dependent on external factors such as temperature and time of the process. Optimal conditions of complete mineralization were established. Both *Fusarium* species were able to biodegrade every isomer of tested compound at 30°C, but *F. culmorum* required 10 days and *F. oxysporum* 11 days to accomplish the process, which was continuously monitored using the ^31^P NMR technique.

## 1. Introduction

Organophosphonates are compounds characterized by the presence of a carbon atom covalently bound to a phosphorus atom. Such compounds are very stable and resistant to thermolysis, chemolysis, photolysis, and biochemical decomposition. [1] Phosphonic acids and their derivatives are molecules of interest due to their structural differentiation and great industrial importance. They are used as crop protection agents (weed control) in water treatment, in metal processing, and as flame-proofing agents [1]. The very valuable group of phosphonate derivatives are hydroxyphosphonates because they are known for their varied biological activities [2]. They act as enzyme inhibitors, antibacterial and antifungal factors, herbicides, antitumor or antiviral drugs, and mostly as molecules of defined absolute configuration. The use of such compounds applies in many consequences, mostly because of the extreme stability of mentioned P-C bond and the steric structure of the molecule. Increasing concentration of phosphonates derivatives in the environment has focused the attention of scientists on the problems with utilization of such pollutants. Biodegradation is a method of choice and represents undoubtedly an environmentally friendly solution.

Some of the soil microorganisms have developed the capability to mineralize and biodegrade such molecules in order to acquire energy and nutrients [3–5]. The effectiveness of such degradation is dependent on several factors, including the chemical and steric structure of compounds and environmental conditions of the process. While bacterial degradation of phosphonic compounds has been thoroughly addressed and reviewed [3, 6], only few, recent works have focused on the use of fungal strains for degradation on such compounds [7–10]. This is interesting because there are advantages in using fungi for bioremediation as they possess extracellular enzymes and larger surface area for absorption and their mycelia provide deeper penetration of the environment [11].

In this work, the ability of *Fusarium oxysporum *(DSMZ 2018) and *Fusarium culmorum *(DSMZ 1094) to mineralize 1-hydroxy-2-(ethoxyphenylphosphinyl) acetic acid was studied. This is the first study, which concerns the biodegradation of chiral hydroxy phosphinates. The previously reported bacterial mineralization considered only not-chiral phosphonates as substrates [12]. The present study also allowed for evaluating the correlation between the effectiveness of fungal biodegradation of chosen compound and the process parameters (time and temperature) and, what is important, defining if there are differences in the rate of particular isomers decomposition. There was another important reason justifying these studies, that is to say, if fungi are able to decompose molecules, which are designed as active agents against themselves, for example, pathogenic fungal strains, including *Fusarium* sp.

## 2. Material and Methods

### 2.1. Chemicals

All materials were of the highest purity available (Fluka, POCh, Sigma-Aldrich).

### 2.2. Microorganisms and Culture Conditions

Both fungal strains were purchased in DSMZ collection, *Fusarium oxysporum *(DSMZ 2018) and *Fusarium culmorum *(DSMZ 1094), and were routinely maintained on potato dextrose agar, which provided profuse sporulation suitable for inoculum collection. The fungi were cultured in H1 medium, which consisted of (w/v) 0.5% yeast extract, 3.0% bactopeptone, 0.05%, MgSO_4_ 0.1% NaNO_3_, 3.0% KH_2_PO_4_, and 1.0% olive oil, pH 7.0. Cultures were grown at 130 rpm in a 250 mL Erlenmeyer flask containing 100 mL medium until the mid-log growth phase. Then the biomass was separated by centrifugation (10 min, 10.000 rpm, 25°C), washed twice with distilled water, and used for the biotransformation process.

### 2.3. Substrate Synthesis

1-hydroxy-2-(ethoxyphenylphosphinyl) acetic acid was synthesized according to the following procedure: glyoxylic acid (3.8 g) was mixed with ethyl phenylphosphinate (6.8 g) and triethylamine (5.6 mL) and the mixture was left at RT on the magnetic stirrer. After 24 h, hydrochloric acid (4 mL, 35–38%) and acetone (100 mL) were added to the mixture. The next step included the evaporation of solvents under reduced pressure and dissolution of residue in methanol (20 mL). After ethyl acetate was added to the mixture up to precipitation the solvents were removed again by evaporation under reduced pressure. The purity of the phosphonic compound was analysed by means of the ^31^P NMR technique.

### 2.4. Biotransformation Procedure

The substrate (50 mg in 500 *μ*L of acetone) was added to the flask containing 50 mL of biotransformation medium (distilled water) and then the pH of medium was adjusted to 7.0, using 5 M solution of NaOH. After that, the biocatalyst (15 g of wet fungal biomass) was added to the reaction mixture. Biotransformation flasks were incubated in a rotary shaker (130 rpm) for various periods of time and at varying temperatures dependent on the outcome. After the specified time of the process the biocatalyst was separated by centrifugation (10 min, 10000 rpm) and the supernatant was extracted twice with ethyl acetate and dried over anhydrous magnesium sulphate. After filtration, the organic layer was evaporated under reduced pressure and the product was analysed by means of the ^31^P NMR spectroscopy using quinine as a chiral discriminator.

### 2.5. ^31^P NMR Analysis: Assignment and Quantification of the Biodegradation Progress

Bioconversion products were analysed *via* NMR spectroscopy. Examined samples (products mixture) were resolved in CDCl_3_ with/without the addition of quinine (*n*/*n* = 1 : 1). Quinine acts as the chiral discriminating solvent, which allowed for separating enantiomers of hydroxyphosphinate, which are then observed on ^31^P NMR spectra. Substrate decomposition was assigned as conversion degree calculated from the comparison of ^31^P NMR signals integration before and after the particular experiment.

## 3. Results and Discussion

**Figure 1:**
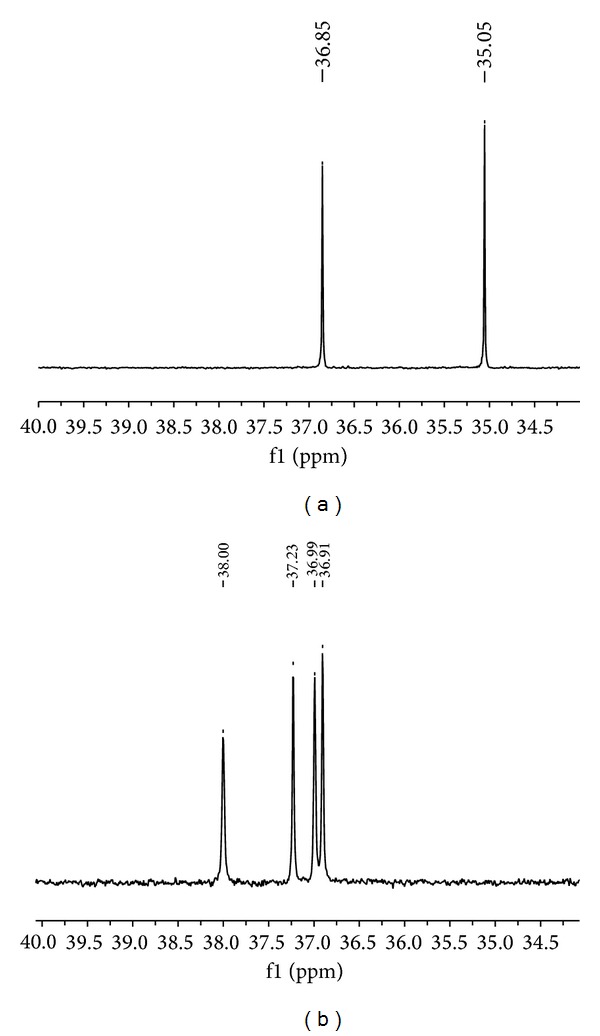
^31^P NMR spectra of substrate. (a) Without quinine; (b) with quinine.

Racemic 2-hydroxy-2-(ethoxyphenylphosphinyl) acetic acid was used as a model substrate in the biocatalytic process catalysed by fungi of the genus *Fusarium* (*Fusarium oxysporum* and *Fusarium culmorum*). The substrate molecule contains two stereogenic centres located at the *α*-carbon atom and at the phosphorus atom, so the racemic mixture consists of four stereoisomers. The application of quinine as a chiral discriminating solvent allowed for visualization of each stereoisomer on the ^31^P NMR spectra (Figure 1) and, as a consequence, also quantification of isomers disappearance after the mineralization was possible.

**Figure 2:**
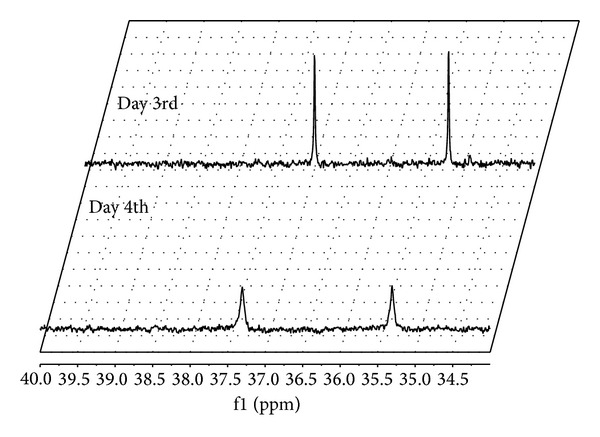
^31^P NMR spectra of postbiotransformation mixtures recorded for *Fusarium oxysporum* used as biocatalysts (recorded without quinine).

The *Fusarium* species are known for their varied enzymatic activities, previously studied by Mandal et al. [13] and Ratuś et al. [14]. Therefore, the whole-cells *Fusarium*-mediated mineralization of diastereoisomers of 2-hydroxy-2-(ethoxyphenylphosphinyl) acetic seemed to be a reasonable approach to complete the utilization of racemic mixture of the starting material. The intent of this work was to elaborate the protocol, which accomplished the process—decomposition of every enantiomeric form of the tested substrate. ^31^P NMR spectra recorded after 3 and 4 days of the process carried out at 25°C confirms the biodegradable activity of chosen fungi. The concentration of the substrate diminished with process time extension for both applied biocatalysts. Representative spectra are shown in Figure 2. After 72 hrs of biotransformation catalysed by *Fusarium oxysporum*, the conversion degree reached 42.2% and after 96 hrs −90.25%.

**Figure 3:**
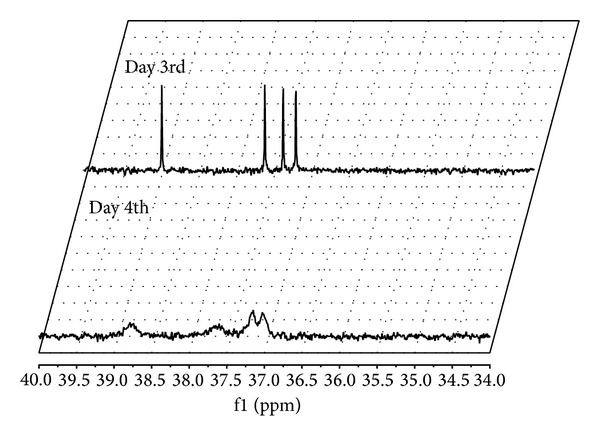
^31^P NMR spectra (with the quinine addition) of postbiotransformation mixtures recorded for *Fusarium oxysporum* used as biocatalysts.

Controlling set of experiments, performed without biocatalysts cells, confirmed that such phosphinate depletion is a consequence of fungal biocatalytic activities (data not shown). This was very promising also because every isomer of the chiral substrate disappeared with a comparable rate, what is sharply indicated on the spectra below performed with the addition of chiral solvating agent (Figure 3: spectra recorded with the addition of quinine).

Discussed preliminary results were an inspiration for the next set of experiments leading to the evaluation of the procedure of complete fungal biodegradation of model substrate.

Subsequent, insightful study of biodegradation of 2-hydroxy-2-(ethoxyphenylphosphinyl) acetate by the *Fusarium* species showed the correlation between external factors (temperature of the process and reaction time) and the effectiveness of the P-C compound utilization. NMR spectra recorded after every outcome allowed for observing intermediates or side products if any appeared. These data proved that any phosphorus containing compound neither organic nor inorganic left or appeared in the examined samples. Such observations lead to conclusions that *Fusarium* species consumed phosphinic compound for cell purposes as a carbon and/or phosphorus source. However, the presence of inorganic Pi derived from C-P compounds mineralized by microorganisms is known from the previous literature data [6].

**Figure 4:**
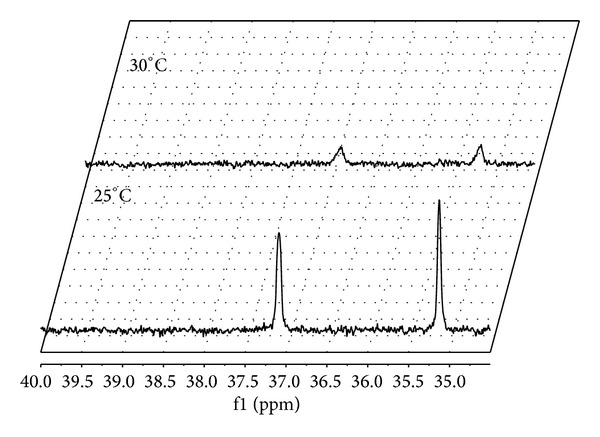
^31^P NMR spectra of postbiotransformation mixtures recorded for *Fusarium culmorum* biocatalysis.

The very significant influence of temperature on the obtained results was noticed. Both biocatalysts became more effective with the temperature increasing from 25°C up to 30°C. The representative results obtained for *Fusarium culmorum* are shown in Figure 4. The raise of biotransformation temperature by 5°C strongly affected the conversion degrees of the substrate—at 25°C the conversion degree was 10.2% and at 30°C this value reached 90.35%—after 120 hours of the process, whereas under the same conditions, the yield of degradation with the use of *Fusarium oxysporum* increased from 65% to 80%. Discussed experiments showed the sensitivity of applied strains to different bioreaction conditions. This is in good agreement with the literature data [15], discussing different ecotypes of *Fusarium.*

**Figure 5:**
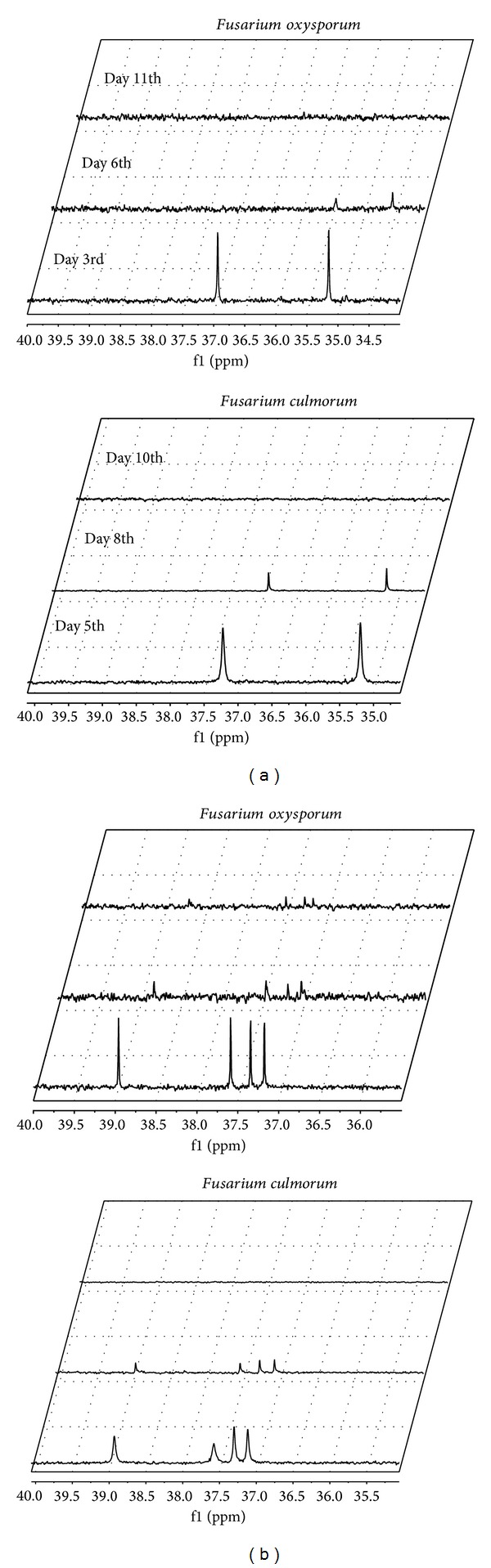
^31^P NMR spectra of postbiotransformation mixtures, recorded for *Fusarium culmorum* and *Fusarium oxysporum* used as biocatalysts in biotransformation carried out under variable time conditions. Results recorded without (a) and with quinine (b).

The effect of reaction time on the conversion of 2-hydroxy-2-(ethoxyphenylphosphinyl) acetic acid is depicted in Figure 5. By extending the time of biotransformation, the complete depletion of substrate from the reaction medium was achieved for both biocatalysts. Additionally, the viability of biocatalysts during the course of the experiments was confirmed (data not shown). Such fungi vitality has been described by Summerell and Leslie [16]; regarding *Fusarium* species, this ability to survive in unfavourable conditions is possible thanks to their capability to produce several types of viable spores (sclerotia, chlamydospores, etc.).

It is noteworthy that the time of complete biodegradation of 2-hydroxy-2-(ethoxyphenylphosphinyl) acetate is quite short, which is a crucial and a relevant feature of such processes regarding their further utility (Table 1).

As it is shown in Table 1, *F. oxysporum *strain was more active toward tested chiral P-C compound—91% of the substrate decomposed within 6 days while the second fungus required a substantially longer period, 8 days, to achieve 79% of substrate conversion. However, both applied microbial cultures accomplish the biodegradation, so undertaken efforts succeeded.

## 4. Conclusions

The *Fusarium *species were found to utilize 2-hydroxy-2-(ethoxyphenylphosphinyl) acetic acid for cell purposes. Discussed results enriched the limited knowledge about biodegradation of P-C compounds by microorganisms of eukaryotic origin and are valuable for bioremediation studies. It must be stressed that the discovered *Fusarium* sp. abilities are, as a matter of fact, very dangerous, because similar phosphonates derivatives are applied against pathogenic kinds of this fungus. Considering this context, current studies are relevant and cannot be ignored.

## Figures and Tables

**Table 1:** Correlation between the biodegradation process duration and its effectiveness.

Microorganisms	Time [h]	Conversion [%]
*F. oxysporum *	72	42.4
	144	91.54
	264	98.48

*F. culmorum *	120	10.02
	192	79.04
	240	100

## References

[1] Nowack B (2003). Environmental chemistry of phosphonates. *Water Research*.

[2] Naydenova ED, Todorov PT, Troev KD (2010). Recent synthesis of aminophosphonic acids as potential biological importance. *Amino Acids*.

[3] McGrath JW, Chin JP, Quinn JP (2013). Organophosphonates revealed: new insights into the microbial metabolism of ancient molecules. *Nature Reviews Microbiology*.

[4] Kamat SS, Williams HJ, Raushel FM (2011). Intermediates in the transformation of phosphonates to phosphate by bacteria. *Nature*.

[5] Mendz GL, Mégraud F, Korolik V (2005). Phosphonate catabolism by Campylobacter spp. *Archives of Microbiology*.

[6] Kononova SV, Nesmeyanova MA (2002). Phosphonates and their degradation by microorganisms. *Biochemistry*.

[7] Klimek M, Lejczak B, Kafarski P, Forlani G (2001). Metabolism of the phosphonate herbicide glyphosate by a non-nitrate-utilizing strain of Penicillium chrysogenum. *Pest Management Science*.

[8] Klimek-Ochab M, Lejczak B, Forlani G (2003). A metal-independent hydrolase from a Penicillium oxalicum strain able to use phosphonoacetic acid as the only phosphorus source. *FEMS Microbiology Letters*.

[9] Klimek-Ochab M, Obojska A, Picco AM, Lejczak B (2007). Isolation and characterization of two new microbial strains capable of degradation of the naturally occurring organophosphonate—Ciliatine. *Biodegradation*.

[10] Forlani G, Klimek-Ochab M, Jaworski J, Picco AM, Lejczak B (2006). Phosphonoacetic acid utilization by fungal isolates: occurrence and properties of a phosphonoacetate hydrolase in some penicillia. *Mycological Research*.

[11] Sagar V, Singh DP (2011). Biodegradation of lindane pesticide by non white-rots soil fungus Fusarium sp. *World Journal of Microbiology and Biotechnology*.

[12] Cook AM, Daughton CG, Alexander M (1978). Phosphorus-containing pesticide breakdown products: quantitative utilization as phosphorus sources by bacteria. *Applied and Environmental Microbiology*.

[13] Mandal D, Ahmad A, Khan MI, Kumar R (2002). Biocatalytic transformation of cyclohexanone by *Fusarium* sp.. *Journal of Molecular Catalysis A*.

[14] Ratuś B, Gładkowski W, Wawrzeńczyk C (2009). Lactones 32 [1]: new aspects of the application of Fusarium strains to production of alkylsubstituted *ɛ*-lactones. *Enzyme and Microbial Technology*.

[15] Hudec K, Muchova D (2010). Influence of temperature and species origin on Fusarium spp. and Microdochium nivale pathogenicity to wheat seedling. *Plant Protection Science*.

[16] Summerell BA, Leslie JF (2011). Fifty years of Fusarium: how could nine species have ever been enough?. *Fungal Diversity*.

